# Anti-Inflammatory Effect of* Piper attenuatum* Methanol Extract in LPS-Stimulated Inflammatory Responses

**DOI:** 10.1155/2017/4606459

**Published:** 2017-07-25

**Authors:** You Jin Kim, Jeong Deok, Sunggyu Kim, Deok Hyo Yoon, Gi-Ho Sung, Adithan Aravinthan, Seungihm Lee, Mi-nam Lee, Suntaek Hong, Jong-Hoon Kim, Young-Jin Son, Jae Youl Cho

**Affiliations:** ^1^Gyeonggi Science High School for the Gifted, Suwon 16297, Republic of Korea; ^2^Department of Genetic Engineering, Sungkyunkwan University, Suwon 16419, Republic of Korea; ^3^Research and Business Foundation, Sungkyunkwan University, Suwon 16419, Republic of Korea; ^4^Institute for Bio-Medical Convergence, International St. Mary's Hospital and College of Medicine, Catholic Kwandong University, Incheon, Republic of Korea; ^5^College of Veterinary Medicine, Chonbuk National University, Iksan 54596, Republic of Korea; ^6^Department of Food and Nutrition, Yeonsung University, Anyang 14011, Republic of Korea; ^7^Department of Biochemistry, Lee Gil Ya Cancer and Diabetes Institute, Gachon University, Incheon 21999, Republic of Korea; ^8^Department of Pharmacy, Sunchon National University, Suncheon 57922, Republic of Korea

## Abstract

*Piper attenuatum *is used as a traditional medicinal plant in India. One of the substances in* P. attenuatum* has been suggested to have anti-inflammatory effects. However, there is insufficient research about the anti-inflammatory mechanisms of action of* P. attenuatum*. The effects of* P. attenuatum* methanol extract (Pa-ME) on the production of inflammatory mediators nitric oxide (NO) and prostaglandin E_2_ (PGE_2_), the expression of proinflammatory genes, the translocation level of transcription factors, and intracellular signaling activities were investigated using macrophages. Pa-ME suppressed the production of NO and PGE_2_ in lipopolysaccharide- (LPS-), pam3CSK4-, and poly(I:C)-stimulated RAW264.7 cells without displaying cytotoxicity. The mRNA expression levels of inducible NO synthase (iNOS) and cyclooxygenase 2 (COX-2) were decreased by Pa-ME. P-ME reduced the translocation of p50/NF-*κ*B and AP-1 (c-Jun and c-Fos), as well as the activity of their upstream enzymes Src, Syk, and TAK1. Immunoprecipitation analysis showed failure of binding between their substrates, phospho- (p-) p85 and p-MKK3/6. p-p85 and p-MKK3/6, which were induced by overexpression of Src, Syk, and TAK1, were also reduced by Pa-ME. Therefore, these results suggest that Pa-ME exerts its anti-inflammatory effects by targeting Src and Syk in the NF-*κ*B signaling pathway and TAK1 in the AP-1 signaling pathway.

## 1. Introduction

Inflammation is a kind of innate immunity that provides a defense against pathogens, damaged cells, and other dangerous molecules. Inflammatory cells, such as macrophages and neutrophils, let leukocytes and plasma components come to sites where infection or injury has occurred during inflammation to eliminate dangers [1, 2]. Although inflammation is important to the immune system, excessive activity of inflammatory cells can cause cancer, rheumatoid arthritis, multiple sclerosis, chronic asthma, psoriasis, and other diseases [1, 3, 4]. To treat those inflammation-driven diseases, inflammatory responses should be controlled. Nuclear factor- (NF-) *κ*B and activator protein- (AP-) 1 are two main transcription factors that initiate inflammation by activation of a series of intracellular signals composed of inhibitor of *κ*B kinase (IKK*α*/*β*), AKT (protein kinase B), Src, and Syk for NF-*κ*B and extracellular signal-regulated kinase (ERK), p38, c-Jun N-terminal kinase (JNK), interleukin-1 receptor-associated kinase 1 (IRAK1), IRAK4, transforming growth factor beta-kinase 1 (TAK1), mitogen-activated protein kinase kinase 3 (MKK3), and MKK6 for AP-1 [5–8]. These molecules are usually considered targets for suppression of the NF-*κ*B or AP-1 pathways to obtain anti-inflammatory outcomes.

*Piper attenuatum* is a plant in the Piperaceae family that inhabits the eastern tropical Himalayas, Assam, Khasi Hills, and the Nilgiris in India. The whole plant is used to cure headache and muscular pain [9].* Piper attenuatum* has antibacterial [10] and antioxidant effects [11], and neolignans in* P. attenuatum* fruit are candidate free radical scavengers [12]. In addition, many substances such as cepharanone B, piperolactam A, piperolactam D, and cepharadione A have been isolated from* P. attenuatum *[13]. Cepharanone and piperolactam exhibit anti-inflammatory effects [13]. However, there is no detailed research regarding the anti-inflammatory mechanism of* P. attenuatum*. In this study, we aimed to explore the molecular mechanism of* P. attenuatum* with its methanol extract (Pa-ME) with respect to the NF-*κ*B and AP-1 signaling pathways.

## 2. Materials and Methods

### 2.1. Materials

Methanol extraction of* P. attenuatum* (Pa-ME) was purchased from the Plant Extract Bank in the Plant Diversity Research Center (Daejeon, Republic of Korea; http://extract.kribb.re.kr, e-mail: mplantext@kribb.re.kr). RAW264.7 cells, a transformed macrophage cell line derived from the BALB/c mouse (ATCC number TIB-71), were purchased from ATCC (Rockville, MD, USA). Dimethyl sulfoxide (DMSO), L-N^G^-nitroarginine methyl ester (L-NAME), indomethacin, lipopolysaccharide (LPS,* Escherichia coli* 0111:B4), pam3CSK, and (3-4,5-dimethylthiazol-2-yl)-2,5-diphenyltetrazolium bromide (MTT) were purchased from Sigma Chemical Co. (St Louis, MO, USA). Poly I:C was obtained from Calbiochem (La Jolla, CA). The enzyme immune assay (EIA) kits used to quantitate the levels of PGE_2_ were purchased from Amersham (Little Chalfont, Buckinghamshire, UK). Specific PCR primers for iNOS, TNF-*α*, COX-2, and GAPDH were synthesized from Bioneer Inc. (Daejeon, Republic of Korea). Antibodies that specify phosphorylated and total forms of p65, p50, c-Jun, c-Fos, Lamin A/C, I*κ*B*α*, IKK*α*/*β*, AKT, Src, Syk, ERK, p38, JNK, IRAK1, IRAK4, TAK1, MKK3/6, and *β*-actin were obtained from Cell Signaling (Beverly, MA, USA).

### 2.2. Animals

Male C57BL/6 mice (6–8 weeks old, 17–21 g) were obtained from DAEHAN BIOLINK (Chungbuk, Republic of Korea) and were housed in groups of 6–8 mice under a 12-hour light/dark cycle (lights on at 6 a.m.). Water and pellet diets (Samyang, Daejeon, Republic of Korea) were supplied ad libitum. Animals were cared for in accordance with the guidelines issued by the National Institute of Health for the Care and Use of Laboratory Animals (NIH Publication 80-23, revised in 1996). Studies were performed in accordance with guidelines established by the Institutional Animal Care and Use Committee at Sungkyunkwan University.

### 2.3. Preparation of Peritoneal Macrophages

To obtain peritoneal macrophages, we used C57BL/6 male mice lavaged 4 days after intraperitoneal injection of 1 ml of sterile 4% thioglycollate broth (Difco Laboratories, Detroit, MI). Then, peritoneal macrophages (1 × 10^6^ cells/ml) were washed using RPMI1640 medium with 10% FBS and were plated in 100 mm tissue culture dishes for 4 h at 37°C in 5% CO_2_ in a humidified incubator.

### 2.4. Cell Culture

RAW264.7 cells, HEK293 cells, and peritoneal macrophages were cultured or maintained in RPMI1640 medium with 10% heat-inactivated FBS, 2 mM of L-glutamine, and antibiotics (100 U/ml of penicillin and 100 *μ*g/ml streptomycin). RAW264.7 cells (2 × 10^6^ cells/ml) were incubated at 37°C in a 5% CO_2_, humidified incubator (Heraeus BB15, Thermo Fisher Scientific, Waltham, MA, USA). For experiments, RAW264.7 cells were detached from the plate with a cell scraper.

### 2.5. NO and PGE_2_ Production Assay

LPS, pam3CSK4, and poly(I:C) were added to RAW264.7 cells or peritoneal macrophages and incubated for 24 h after a 30 min Pa-ME pretreatment. 100 *μ*l of supernatant was obtained and mixed with 100 *μ*l of Griess reagent, as reported previously [14]. The absorbance of this mixture was measured at 540 nm. The concentration of NO was calculated using an NO standard. L-NAME was used as a positive control. The effect of Pa-ME on PGE_2_ production was determined by EIAs, as described previously [15].

### 2.6. Cell Viability Assay

Pa-ME was added to testing cells (HEK293 cells, peritoneal macrophages, and RAW264.7 cells) and incubated for 24 h. Then, 10 *μ*l MTT solution (10 mg/ml in PBS pH 7.4) was added and incubated for 3 h as reported previously [16]. The reaction was stopped by 15% sodium dodecyl sulphate and the samples were incubated for another 24 h. The percentage of living cells relative to the control was calculated by using absorbance at 570 nm.

### 2.7. High-Performance Liquid Chromatography (HPLC)

High-performance liquid chromatography (HPLC) analysis was utilized for confirmation of the phytochemical characteristics of Pa-ME with the standard compounds quercetin, luteolin, and kaempferol [17]. The analysis used a system equipped with a KNAUER (Wellchrom) HPLC-pump K-1001, a Wellchrom fast scanning spectrophotometer K-2600, and a 4-channel degasser K-500. Elution solvents were solvent A (0.1% H_3_PO_4_ in H_2_O) and solvent B (acetonitrile). The gradient step of the solvent was solvent A to solvent B/min, and a Phenomenex Gemini C_18_ ODS (5 *μ*m) column was used.

### 2.8. Measurement of mRNA Expression Levels by Reverse-Transcriptase Polymerase Chain Reaction (RT-PCR)

To measure the expression level of mRNA related to inflammatory signaling, RT-PCR was conducted. RAW264.7 cells were incubated for 6 h with LPS (1 *μ*g/ml) after 30 min of Pa-ME pretreatment. Total RNA was obtained using TRIzol reagent (Gibco BRL) according to the manufacturer's instructions. Total mRNA was frozen at −70°C for future use. First, 1 *μ*g of RNA was incubated at 70°C with oligo-dT for 5 minutes. After that, it was incubated for 5 more minutes after mixing with 5x first-strand buffer, 10 mM dNTPs, and 0.1 M dithiothreitol (DTT). Then, we added MuLV reverse-transcriptase (2 U) and incubated the mixture at 37°C for 60 min and 70°C for 10 minutes. The remaining RNAs were removed by RNase H. The polymerase chain reaction (PCR) was conducted with the incubation mixture (2 *μ*l cDNA, 1 *μ*l 5′ primer, 1 *μ*l 3′ primer, and 6 *μ*l diethyl pyrocarbonate (DEPC)) in a 10 *μ*l premix using the RT-thermal cycler (Bio-Rad, Hercules, CA, USA). The primer (Bioneer, Daejeon, Republic of Korea) sequence is listed in Table 1. Amplified cDNA was loaded onto a 1.5% agarose gel with TAE buffer with 0.5 *μ*g/ml ethidium bromide (EtBr) and run at 100 V for 1 h. The relative quantities were visualized with the DNR Bio-imaging system (Jerusalem, Israel).

### 2.9. Preparation of Nuclear/Total Lysates of Cells

RAW264.7 cells were washed with cold PBS containing 1 mM sodium orthovanadate and lysed using a sonicator (Thermo Fisher Scientific, Waltham, MA, USA) in ice-cold modified RIPA buffer (50 mM Tris-HCl (pH 7.4), 1% Nonidet P-40, 0.25% sodium deoxycholate, 150 mM NaCl, 1 mM Na_3_VO_4_, and 1 mM NaF) containing protease inhibitors (2 mM PMSF, 100 *μ*g/ml leupeptin, 10 *μ*g/ml pepstatin, 1 *μ*g/ml aprotinin, and 2 mM EDTA) for 30 min with rotation at 4°C. Lysates were refined by centrifugation at 16,000 ×g for 10 minutes at 4°C and stored at −20°C until use. Nuclear fractions were prepared in a three-step procedure. First, after treatment with Pa-ME, we collected cells with a rubber policeman and lysed them with 500 ml lysis buffer (50 mM KCl, 0.5% Nonidet P-40, 25 mM HEPES, 1 mM phenylmethylsulfonyl fluoride, 10 *μ*g/ml leupeptin, 20 *μ*g/ml aprotinin, and 100 *μ*M 1,4-dithiothreitol) on ice for 4 min. Lysates were centrifuged at 16000 ×g for 1 min. Second, the pellet was washed with the washing buffer (lysis buffer without Nonidet P-40). Finally, we incubated the nuclei with extraction buffer (lysis buffer with 500 mM KCl and 10% glycerol), froze the nuclei/extraction buffer mixture at −80°C, and centrifuged the mixture at 16000 ×g for 5 minutes. The supernatant was collected as a nuclear extract. Soluble cell lysates (30 *μ*g/lane) were immunoblotted.

### 2.10. Detection of Proteins by Western Blot Analysis

The phosphorylated or total levels of p65, p50, c-Jun, c-Fos, Lamin A/C, I*κ*B*α*, IKK*α*/*β*, AKT, Src, Syk, ERK, p85, p38, JNK, IRAK1, IRAK4, TAK1, MKK3, MKK3/6, and *β*-actin were visualized by Western blotting. Nuclear and whole cell extracts containing protein were subjected to 7–15% sodium sulphate polyacrylamide gel electrophoresis (SDS-PAGE). Then, the gel was transferred onto a polyvinylidene difluoride (PVDF) membrane and blocked using BSA. The membrane was rotated overnight with the first antibody in BSA. It was washed with Tris-buffered saline with Tween 20 (TBST) and then probed with a second antibody conjugated with horseradish peroxidase in BSA for 1 h. Using an enhanced chemiluminescence kit (Pierce ECL Western blotting substrate, Thermo Scientific, Waltham, MA, USA), immunoreactive bands were detected.

### 2.11. Immunoprecipitation

Cell lysates containing equal amounts of protein (500 *μ*g) from RAW264.7 cells (1 × 10^7^ cells/ml) treated with or without LPS (1 *μ*g/ml) for 3 min were precleared with 10 *μ*l protein A-coupled Sepharose beads (50% v/v) (Amersham, UK) for 1 h at 4°C, as reported previously [18]. Then, the samples were incubated with 5 *μ*l antibodies to Syk, Src, or TAK1 overnight at 4°C. The antigen-antibody complexes were mixed with 10 *μ*l protein A-coupled Sepharose beads (50% v/v) and rotated for 3 h at 4°C. After that, the immunoprecipitates were analyzed by immunoblotting analysis.

### 2.12. Plasmid Transfection and Luciferase Reporter Assay

HEK293T cells (1 × 10^6^ cells/ml) were transfected with 1 *μ*g of HA-TAK1 alone or plasmids containing *β*-galactosidase (*β*-Gal) (as a control) and AP-1-Luc in the presence or absence of an inducing molecule, TAK1. Transfections were performed using the PEI method in 6-well plates, as previously outlined [19, 20]. Transfected cells were used at 48 h after transfection for all experiments. Cells were treated with Pa-ME for the final 24 h of each experiment. Luciferase assays were performed using the Luciferase Assay System (Promega), as previously reported [21].

### 2.13. Statistical Analysis

**Figure 1:**
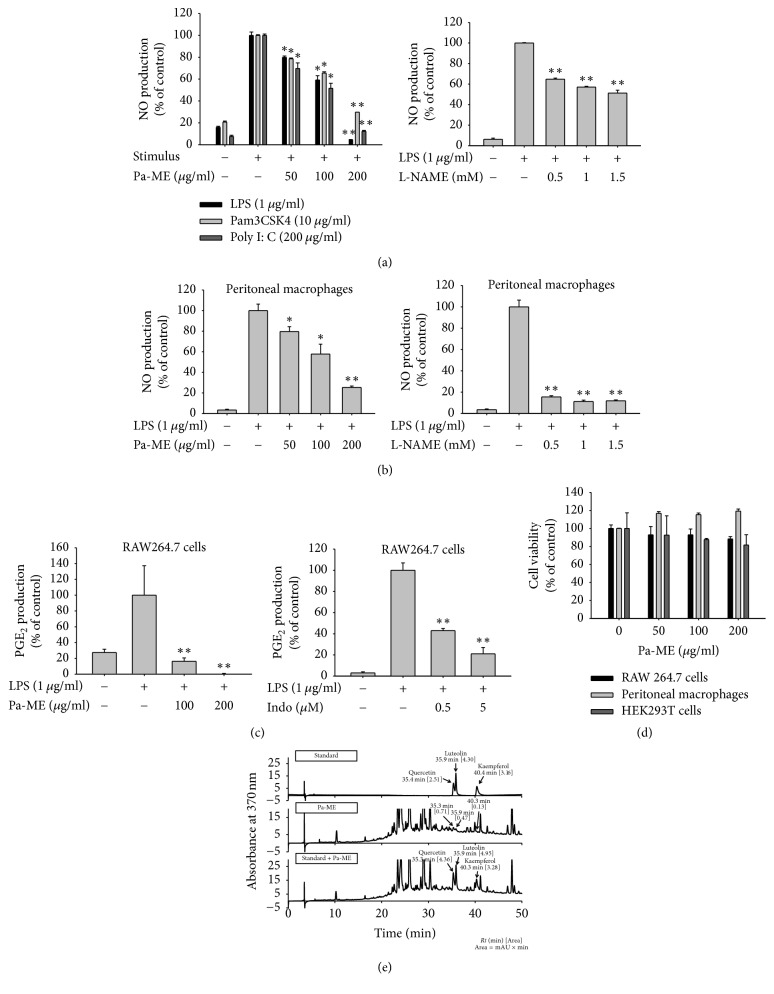
Effect of Pa-ME on NO production and cell viability. (a, b, and c) Cells were pretreated with Pa-ME or standard compounds [Indo (indomethacin) and L-NAME] and incubated for 24 h with TLR ligands (LPS, pam3CSK4, and poly(I:C)). The levels of NO (a and b) and PGE_2_ (c) were analyzed by Griess assay or EIA from the culture supernatant of RAW264.7 cells (a and c) or peritoneal macrophages (b) which were stimulated by LPS (1 *μ*g/ml), poly(I:C) (200 *μ*g/ml), and pam3CSK4 (10 *μ*g/ml) (right panel) in the presence or absence of Pa-ME or L-NAME. (d) Viability of RAW264.7 cells (left panel) or peritoneal macrophages (right panel) was determined by MTT assay. (e) The phytochemical profile of Pa-ME was analyzed by HPLC with standard flavonoids, quercetin, luteolin, and kaempferol [Area]. ^*∗*^*P* < 0.05 and ^*∗∗*^*P* < 0.01 compared with control group.

Data presented herein are the mean ± SD of in vitro experiments performed with two samples (Figures 1(a) and 1(b)). For statistical comparisons, results were analyzed using analysis of variance/Scheffe's post hoc test and the Kruskal-Wallis/Mann-Whitney test. A *P* value < 0.05 was considered statistically significant. All statistical tests were carried out using SPSS software (SPSS Inc., Chicago, IL, USA).

## 3. Results

### 3.1. Pa-ME Suppressed the Production of NO

Since NO is a representative mediator of inflammation, we examined whether Pa-ME was able to suppress inflammatory responses by determining NO levels in culture supernatant prepared from activated RAW264.7 cells in the presence or absence of Pa-ME (0 to 200 *μ*g/ml). As Figure 1(a) shows, the amount of NO was enhanced via treatment with inflammation inducers (LPS, pam3CSK4, and poly(I:C)) and was dose-dependently decreased by Pa-ME (left panel), as in the case of the standard NO inhibitor, L-NAME (right panel). Dose-dependent NO inhibition by Pa-ME and L-NAME in a cancerous macrophage cell line was also exhibited in primary cells (peritoneal macrophages) (Figure 1(b)). Moreover, this extract (100 and 200 *μ*g/ml) downregulated the release of PGE_2_ as shown in the case of COX inhibitor, indomethacin (Figure 1(c)). Viability measurement of RAW264.7 cells, HEK293 cells, and peritoneal macrophages (right panel) treated with Pa-ME revealed that Pa-ME inhibition of NO was not due to nonspecific suppression of cell viability (Figure 1(d)). Meanwhile, to check the phytochemical profile of Pa-ME, we performed high-performance liquid chromatography (HPLC) analysis of Pa-ME with flavonoids such as quercetin, luteolin, and kaempferol, which are known to inhibit NO release [18, 22]. As Figure 1(e) and Table 2 show, quercetin, luteolin, and kaempferol were detected at 35.3, 35.9, and 40.3 min at 0.004, 0.001, and 0.001%, respectively.

### 3.2. Pa-ME Suppressed Inflammatory Gene Expression and Nuclear Translocation of NF-*κ*B and AP-1

**Figure 2:**
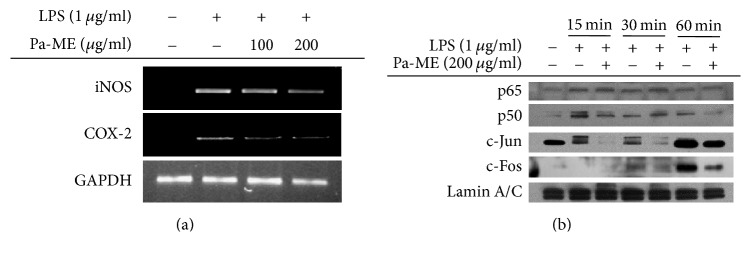
Effect of Pa-ME on the transcriptional activation of proinflammatory genes. (a) Levels of iNOS, COX-2, and GAPDH mRNA were determined by semiquantitative PCR. (b) Nuclear levels of NF-*κ*B (p65 and p50), AP-1 (c-Jun and c-Fos), and Lamin A/C in nuclear fractions were determined by nuclear fractionation and immunoblotting analysis.

The effect of Pa-ME on inflammatory gene expression was examined by analysis of iNOS and COX-2 mRNA levels by reverse-transcription polymerase chain reaction. As Figure 2(a) depicts, the quantity of iNOS and COX-2 was dose-dependently reduced by Pa-ME. To confirm whether this extract was capable of modulating the activation of transcription factors, the nuclear translocation levels of NF-*κ*B and AP-1 were detected by Western blotting with nuclear fractions. As Figure 2(b) indicates, the nuclear level of p50/NF-*κ*B was reduced by Pa-ME at 15 and 60 min of LPS treatment. In addition, c-Jun and c-Fos, major AP-1 subunits, were also suppressed by Pa-ME at 15, 30, and 60 min (Figure 2(b)).

### 3.3. Pa-ME Suppresses Upstream Proteins in NF-*κ*B Signaling Pathway

**Figure 3:**
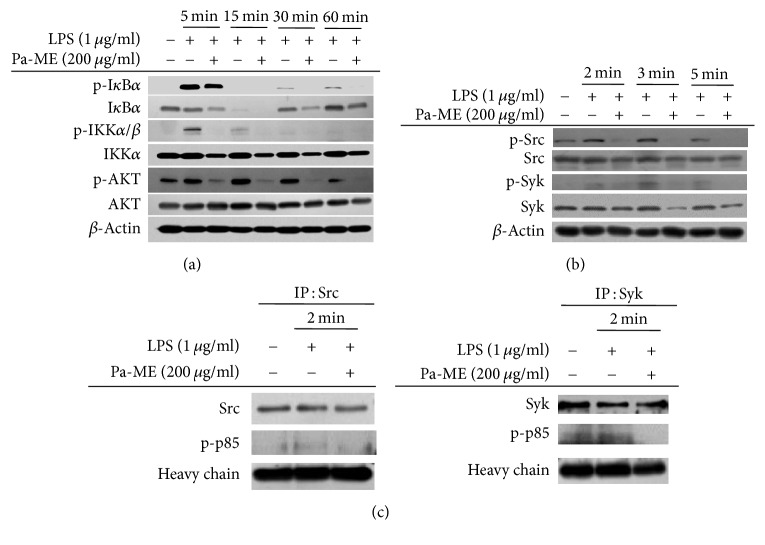
Effect of Pa-ME on the activation of upstream signaling molecules for NF-*κ*B translocation. (a and b) Phosphorylated or total forms of I*κ*B*α*, IKK*α*/*β*, AKT, Src, Syk, and *β*-actin levels at 5, 15, 30, and 60 min or 2, 3, and 5 min were detected by immunoblotting analysis with phospho-specific or total protein antibodies from total cell lysates. (c) The binding level of p-p85 to Src (left panel) or Syk (right panel) at 2 min was detected by immunoprecipitation and immunoblotting analysis of LPS-treated RAW264.7 cells in the presence or absence of Pa-ME.

To verify the immunopharmacological targets of Pa-ME, we elucidated the upstream signaling molecules that were involved in the NF-*κ*B signaling pathway immunoblotting analysis. Interestingly, the phosphorylated forms of I*κ*B*α*, IKK*α*/*β*, and AKT were reduced by Pa-ME at 30 and 60 min after LPS treatment, while their total forms remained constant (Figure 3(a)). Moreover, the phosphorylation levels of the upstream protein tyrosine kinases, Src and Syk, were also decreased at 2, 3, and 5 min without alteration of their total levels (Figure 3(b)). To ensure that these proteins could be targeted by Pa-ME, we further conducted immunoprecipitation analysis to measure the binding levels of p-p85, an active substrate of Src, Syk, and p85. The binding level of phospho-p85 was clearly reduced in the immunoprecipitation mixture composed of Syk or Src after treatment with Pa-ME (Figure 3(c)).

### 3.4. Pa-ME Suppresses Upstream Proteins in the AP-1 Signaling Pathway

**Figure 4:**
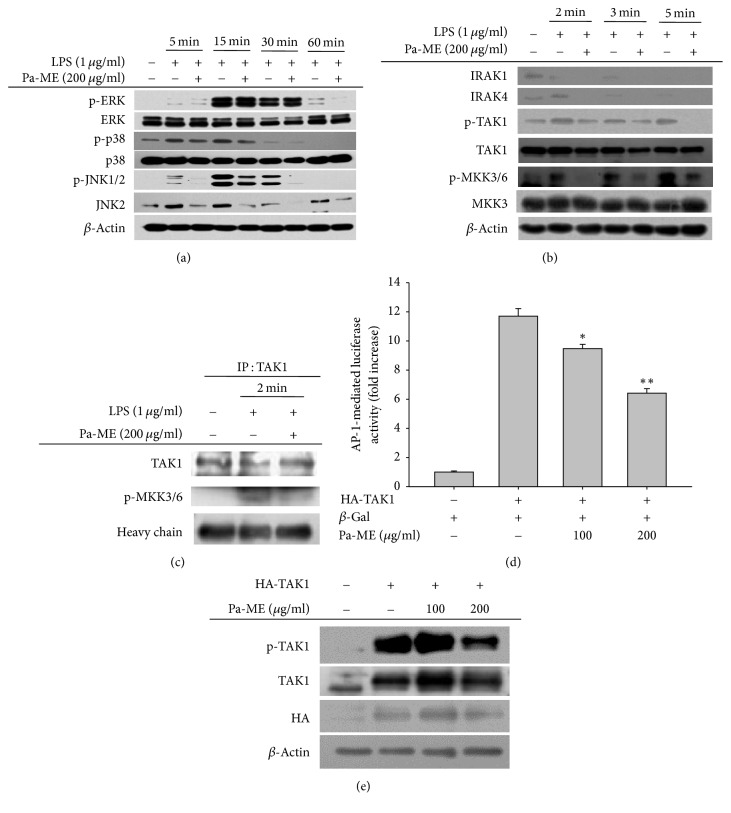
Effects of Pa-ME on the activation of upstream signaling molecules for AP-1 translocation. (a and b) Phosphorylated or total forms of ERK, p38, JNK1/2, IRAK1, IRAK4, TAK1, MKK3/6, MKK3, and *β*-actin levels at 5, 15, 30, and 60 min or 2, 3, and 5 min were detected by immunoblotting analysis with phospho-specific or total protein antibodies from total cell lysates. (c) The binding level of p-MKK3/6 to TAK1 at 2 min was detected by immunoprecipitation and immunoblotting analysis of LPS-treated RAW264.7 cells in the presence or absence of Pa-ME. *β*-Gal, *β*-galactosidase. ^*∗*^*P* < 0.05 and ^*∗∗*^*P* < 0.01 compared with control group.

We evaluated whether Pa-ME could modulate the AP-1 activation pathway by measuring MAPK protein phosphorylation. As Figure 4(a) shows, the phosphorylation levels of ERK, p38, and JNK1/2 and their upstream enzymes MKK3/6 and TAK1 under LPS stimulation at 2, 3, and 5 min were decreased by Pa-ME (Figure 4(b)). Since TAK1 is upstream of the MAPK/AP-1 pathway, a further validation experiment was carried out by immunoprecipitation analysis. Interestingly, the binding of p-MKK3/6 to TAK1 was clearly reduced by Pa-ME treatment (Figure 4(c)). In addition, the luciferase activity induced by TAK1/AP-1 was also dose-dependently suppressed by this extract (Figure 4(d)), according to reporter gene assay. Moreover, Pa-ME suppressed the phosphorylation of TAK1 triggered under HA-TAK1 overexpression (Figure 4(e)).

## 4. Discussion

NO, which stands for nitric oxide, is secreted as a result of inflammation and is involved in the innate response and cytotoxicity [23]. LPS, pam3CSK4, and poly(I:C) are the ligands of toll-like receptors TLR4, TLR2/TLR1, and TLR3, respectively [24], so they can trigger inflammatory responses. When we measured the amount of NO after Pa-ME pretreatment and treatment with LPS, pam3CSK4, and poly(I:C) in RAW264.7 cells, we found that Pa-ME suppressed NO and PGE_2_ production in a dose-dependent manner (Figures 1(a) left panel, and 1(c) left panel) with no significant effect on cell viability in the 50–200 *μ*g/ml concentration range (Figure 1(d)). When we changed the cell type into peritoneal macrophages, similar data were produced (Figures 1(b) left panel, and 1(c) right panel). Since Pa-ME and L-NAME have similar effects on the nitric oxide synthase inhibitor, it appears that Pa-ME suppresses NO production. By analyzing the phytochemical properties of Pa-ME with HPLC (Figure 1(e) and Table 2), we identified flavonoids such as quercetin, luteolin, and kaempferol, which were known for their anti-inflammatory effects [25–27]. There were peaks corresponding to the retention time of the standard compound, and combined samples with Pa-ME and flavonoids enhanced the area proportionally (Figure 1(e)). This ensures that Pa-ME can decrease NO production in inflammatory situations.

Under inflammatory conditions, NO is generated by iNOS in macrophages [28]. Various genes in addition to iNOS are used to make cytokines that mediate inflammation. Representative inflammatory mRNA, iNOS, and COX-2 levels were examined in this study, and Pa-ME reduced iNOS and COX-2 (Figure 2(a)). Thus, iNOS and COX-2 were decreased in a dose-dependent manner at 0 to 200 *μ*g/ml. Therefore, the ideal concentration of Pa-ME for inhibiting inflammation is 200 *μ*g/ml, and we used this concentration in experiments afterwards. It is generally known that NF-*κ*B regulates iNOS and AP-1 regulates COX-2 [29]. NF-*κ*B consists of p65 and p50, while AP-1 consists of c-Fos and c-Jun [30, 31]. The subunits are merged in the cytosol and translocate into the nucleus. If Pa-ME directly regulates gene transcription, the nuclear quantities of p65, p50, c-Fos, and c-Jun should not be decreased. However, immunoblotting of the nuclear lysate showed reduced protein levels of p50, c-Jun, and c-Fos (Figure 2(b)), implying that Pa-ME affects upstream signals that regulate transcription factors. First, we confirmed the NF-*κ*B signaling pathway and tried to identify molecules linking to Pa-ME pharmacology. In the NF-*κ*B signaling pathway, I*κ*B*α*, IKK*α*, IKK*β*, AKT, Src, and Syk are involved. By Western blotting, we found that the phospho-forms of I*κ*B*α*, IKK*α*/*β*, and AKT were decreased at 30 and 60 min (Figure 3(a)). In addition, Src and Syk were reduced at 2, 3, and 5 min, and there were few changes in the total forms of all enzymes (Figure 3(b)). Because there is no enzyme upstream of Src and Syk, these may be Pa-ME targets. We further validated this possibility by carrying out immunoprecipitation/immunoblotting analysis and overexpression of these genes. Total Src and Syk did not change with Pa-ME treatment, while this extract reduced the p-p85 levels bound in Src or Syk complexes (Figure 3(c)). Overall, it is likely that Pa-ME might target Src and Syk during its anti-inflammatory activity toward the NF-*κ*B signaling pathway.

We also explored whether Pa-ME could modulate the AP-1 signaling pathway, as the COX-2 mRNA level was decreased (Figure 2(a)). There are three subpathways within the AP-1 signaling pathway which are composed of JNK, p38, and ERK [32]. Interestingly, Pa-ME reduced the phosphorylation of all these MAPKs (Figure 4(a)), implying that some common protein(s) might be targeted by this extract. By evaluating common upstream molecules, we identified that TAK1 might be a Pa-ME target for suppression of the AP-1 pathway. Indeed, the binding of p-MKK3/6 to TAK1 was reduced by treatment with this extract (Figure 4(c)). TAK1 overexpression-derived phosphorylation and luciferase activity were also effectively suppressed by Pa-ME (Figures 4(d) and 4(e)). These results therefore seem to suggest that Pa-ME also targets the TAK1 enzyme to modulate the AP-1 pathway.

**Figure 5:**
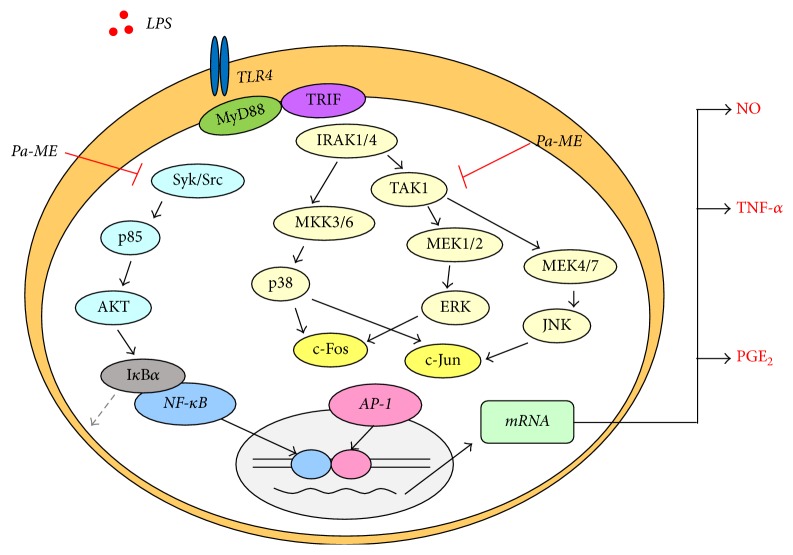
Putative inhibitory pathway of Pa-ME-mediated anti-inflammatory responses. Pa-ME suppressed both Syk and Src kinase activity in the NF-*κ*B pathway and TAK1 kinase activity in the AP-1 pathway. Following that, Pa-ME inhibited the activity of downstream molecules, leading to anti-inflammatory effects.

In conclusion, we revealed that Pa-ME can relieve inflammation by inhibiting Src, Syk, and TAK1 to suppress both the NF-*κ*B and AP-1 pathways, as summarized in Figure 5. Although Pa-ME was proposed as a potential anti-inflammatory remedy, our data clearly provide experimental evidence to support its anti-inflammatory activity. Further preclinical studies on anti-inflammatory action using various in vivo models will be needed to confirm the anti-inflammatory properties of this plant for therapeutic use.

## Figures and Tables

**Table 1:** Sequences of primers used for semiquantitative reverse-transcription PCR in this study.

Targets	Sequences (5′ to 3′)
iNOS	
Forward	GGAGCCTTTAGACCTCAACAGA
Reverse	TGAACGAGGAGGGTGGTG
COX-2	
Forward	GGGAGTCTGGAACATTGTGAA
Reverse	GCACATTGTAAGTAGGTGGACTGT
GAPDH	
Forward	GCACATTGTAAGTAGGTGGACTGT
Reverse	AGGGAGATGCTCAGTGTTGG

**Table 2:** The estimated content of anti-inflammatory flavonoids in Pa-ME.

Flavonoid	Content	
	mg/g	%
Quercetin	0.038	0.004
Luteolin	0.010	0.001
Kaempferol	0.015	0.001

## Abbreviations

NO: Nitric oxide
LPS: Lipopolysaccharide
Pam3CSK4: Synthetic bacterial lipopeptide Pam3Cys-Ser-(Lys)4
Poly(I:C): Polyinosinic:polycytidylic acid
L-NAME: L-N^G^-nitroarginine methyl ester
MTT: 3-(4,5-Dimethylthiazol-2-yl)-2,5-diphenyltetrazolium bromide
HPLC: High-performance liquid chromatography
RT-PCR: Reverse-transcriptase polymerase chain reaction
iNOS: Inducible NO synthase
COX-2: Cyclooxygenase-2
TNF-*α*: Tumor necrosis factor-alpha
IP: Immunoprecipitation
Src: Nonreceptor tyrosine kinase
Syk: Spleen tyrosine kinase
TAK1: Transforming growth factor beta-kinase 1.
